# Maintaining Vaccine Delivery Following the Introduction of the Rotavirus and Pneumococcal Vaccines in Thailand

**DOI:** 10.1371/journal.pone.0024673

**Published:** 2011-09-13

**Authors:** Bruce Y. Lee, Tina-Marie Assi, Korngamon Rookkapan, Angela R. Wateska, Jayant Rajgopal, Vorasith Sornsrivichai, Sheng-I Chen, Shawn T. Brown, Joel Welling, Bryan A. Norman, Diana L. Connor, Rachel R. Bailey, Anirban Jana, Willem G. Van Panhuis, Donald S. Burke

**Affiliations:** 1 University of Pittsburgh, Pittsburgh, Pennsylvania, United States of America; 2 Prince of Songkla University, Hat Yai, Songkhla, Thailand; 3 Pittsburgh Supercomputing Center, Pittsburgh, Pennsylvania, United States of America; Yale University, United States of America

## Abstract

Although the substantial burdens of rotavirus and pneumococcal disease have motivated many countries to consider introducing the rotavirus vaccine (RV) and heptavalent pneumococcal conjugate vaccine (PCV-7) to their National Immunization Programs (EPIs), these new vaccines could affect the countries' vaccine supply chains (i.e., the series of steps required to get a vaccine from their manufacturers to patients). We developed detailed computational models of the Trang Province, Thailand, vaccine supply chain to simulate introducing various RV and PCV-7 vaccine presentations and their combinations. Our results showed that the volumes of these new vaccines in addition to current routine vaccines could meet and even exceed (1) the refrigerator space at the provincial district and sub-district levels and (2) the transport cold space at district and sub-district levels preventing other vaccines from being available to patients who arrive to be immunized. Besides the smallest RV presentation (17.1 cm^3^/dose), all other vaccine introduction scenarios required added storage capacity at the provincial level (range: 20 L–1151 L per month) for the three largest formulations, and district level (range: 1 L–124 L per month) across all introduction scenarios. Similarly, with the exception of the two smallest RV presentation (17.1 cm^3^/dose), added transport capacity was required at both district and sub-district levels. Added transport capacity required across introduction scenarios from the provincial to district levels ranged from 1 L–187 L, and district to sub-district levels ranged from 1 L–13 L per shipment. Finally, only the smallest RV vaccine presentation (17.1 cm^3^/dose) had no appreciable effect on vaccine availability at sub-districts. All other RV and PCV-7 vaccines were too large for the current supply chain to handle without modifications such as increasing storage or transport capacity. Introducing these new vaccines to Thailand could have dynamic effects on the availability of all vaccines that may not be initially apparent to decision-makers.

## Introduction

Although the substantial burdens of rotavirus and pneumococcal disease have motivated many countries to consider introducing the rotavirus vaccine (RV) and heptavalent pneumococcal conjugate vaccine (PCV-7) to their National Immunization Programs (EPIs), these new vaccines could affect the countries' vaccine supply chains (i.e., the series of steps required to get a vaccine from the manufacturers to the arms and mouths of patients). Vaccine vials can come in different presentations, i.e., the physical appearance and composition of the vaccine vial and associated packaging. For example, a vial can contain different numbers of vaccine doses (e.g., a single dose, 2-doses, 10-doses, or 20-doses). Moreover, vaccines can be in liquid or powder form, the latter requiring reconstitution with a diluent (i.e., saline to be mixed with the vaccine to produce a liquid mixture). Presentations can include vaccines alone or bundled or integrated with diluents or delivery devices (e.g., droppers or syringes), which can substantially increase the volume occupied in the supply chain. When determining what vaccines to introduce and vaccine presentations to procure, decision makers may consider the price of the vaccine, disease burden, ease of administration, and existing relationships with manufacturers. However, as previous history has indicated, they may not fully anticipate the new vaccine's potential impact on the supply chain (e.g., the smallest vaccine may not be the least expensive, the easiest to administer, or produced by a manufacturer who already supplies other vaccines to the country).

For example, in 2006–2007 introducing the Rotateq and Rotarix RVs to Latin and South American countries (Brazil, Ecuador, El Salvador, Mexico, Nicaragua, Panama and Venezuela) displaced other existing World Health Organization (WHO) recommended Expanded Programs on Immunization (EPI) vaccines from already limited refrigerator and transport space [1], [2]. Merck's RotaTeq and GlaxoSmithKline's Rotarix were too large for many of the existing supply chains, especially at the periphery, in Latin America. Many clinics did not have sufficient refrigerator capacity to accommodate the addition of larger volume vaccines, and health care workers could not carry the extra thermoses and cold boxes needed to transport the vaccines. Vaccine stock-outs occurred frequently, limiting vaccine availability for patients. No training or operational plans were in place to deal with these unanticipated consequences, resulting in the expiration of large stocks of relatively expensive vaccines [1]. Manufacturers were therefore compelled to reduce the packaging size of these vaccines [1], [3].

RV and PCV-7 are high priority vaccine candidates for introduction into Thailand's EPI. After neonatal complications (45%), rotaviruses (16%) and pneumonia (11%) are the two leading causes of under-five mortality in Thailand [4]. The Vaccine Modeling Initiative (VMI), in collaboration with the Southern Vaccine Research Team (SVRT) from the Prince of Songkla University (PSU) in Hat Yai, Thailand, have developed computational models of the Thailand supply chain to forecast the impact of introducing these vaccines. Experiments explored introducing different presentations of the RV and PCV-7 vaccines separately and in various combinations.

## Methods

We developed two models of the vaccine supply chain, representing every storage location, refrigerator, freezer, transport vehicle, and warehouse or clinic:

- Discrete event simulation model, i.e., Highly Extensible Resource for Modeling Event-Driven Simulations (HERMES): A detailed simulation model representing all the processes, storage and administration locations, personnel and equipment (transport, administration, and other), in the Southern Thailand supply chain developed in the program language Python, using features provided by the SimPy package [5].
- Deterministic mathematical equation-based model (EBM): A series of mathematical equations representing the flow of all EPI vaccines from vaccine manufacturers to the Thailand central depot and storage locations at all levels in Southern Thailand to the patients. Development of the EBM occurred in C++ and Microsoft Excel (Microsoft Corporation, Redmond, WA) and optimization in CPLEX (IBM Corporation, Armonk, NY, USA), respectively [5].

### Overview of Trang Province Vaccine Supply Chain

**Figure 1:**
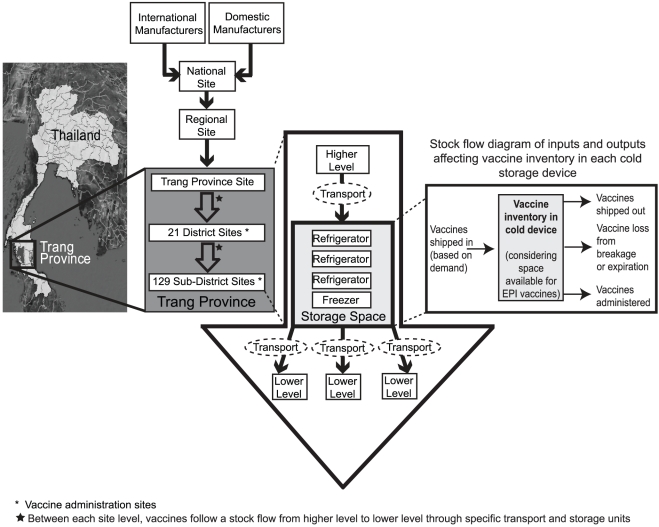
Trang Province Supply Chain Network.

The Thailand vaccine supply chain consists of five levels: national, regional, provincial, district, and sub-district. Our model focuses on the province of Trang (one of seventy six provinces in Thailand) supply chain (Figure 1) [6], which contains 152 locations: 1 regional site (cold storage capacity = 34,660 L), 1 provincial site (660 L of refrigerator space and 1,080 L of freezer space), 21 district sites (3 Municipal Health Centers = 160 L of refrigerator space and 23 L of freezer space, 8 District Health Offices = 24 L of refrigerator space, 16 L of freezer space and 9 Hospitals = 245 L of refrigerator and freezer space each) and one intermediary site, and 129 sub-district sites (160 L of refrigerator space and 23 L of freezer space). Storage capacities and transport capacities came from a sample of selected supply chain locations and are consistent across a level. Data to construct the models came from extensive inventories of and visits to locations at each level as well as the Expanded Program on Immunization (EPI) in association with the Ministry of Public Health (Bangkok, Thailand).

Thailand procures their EPI vaccines from both domestic and international manufacturers. The Government Pharmaceutical Organization (GPO) repackages some vaccines into systematically-sized batches for national distribution and delivers them to regional sites which in turn send the vaccines down the supply chain. The Trang province site receives vaccines monthly and supplies district sites which include hospitals, municipal health centers, and district health offices, and sub-district sites with vaccines on a monthly basis. Each sub-district receives vaccines for their own immunization sessions and for school-based vaccination sessions. Designated vehicles from district and sub-district sites go up to the next level to collect vaccines and bring them back as scheduled. At the district level, immunization sessions occur only once per week, and at the sub-district level, only once per month. This is done to minimize open vial wastage, simplify immunization surveillance, and manage personnel time devoted to the NIP. At hospitals, birth doses are administered as births occur.

### Supply Chain Processes and Policies

Both HERMES and the EBM represent every location (points of distribution and administration) in the Trang province supply chain, including their respective transport and storage capacities. The number of vaccines currently stored in a refrigerator or freezer is tracked as the vaccine inventory. This inventory is equal to the number of vaccines remaining from the previous day minus the vaccines that are shipped, administered (if the location is a district or sub-district), or wasted, plus the number of vaccines that are delivered that day:
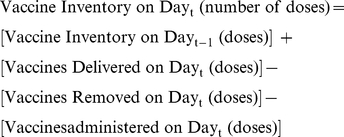
Freezer (−15°C to −25°C) and refrigerator (2°C to 8°C) capacities for each location were pre-defined. The percentage of physical space within the freezer or refrigerator that can actually be used for storage was the utilization rate. This was kept at a constant 85% within the model to account for the space used by other temperature sensitive products in addition to shelving. The total volume of vaccines (the number of each type of vaccine multiplied by its volume per vial) can never exceed the refrigerator or freezer's storage capacity multiplied by its utilization rate. The baseline (current policy) shipping frequency occurs on a monthly schedule wherein shipments from location to location occur at defined frequencies that are specific to the level and transportation route. While EPI policies in Thailand prescribe shipping vaccines monthly below the regional level, some locations may distribute or collect vaccines more or less frequently in a month as needed. The vaccine shipments cannot contain more vaccine vials than the specified storage capacity of that particular transport vehicle or person which translates to a 4×4 vehicle at the provincial level, a cold box at the district level, and a smaller vaccine carrier at the sub-district level.

### Patient Demand and Arrival Rates

The model assumed that children would present to clinics for immunization when they reach the appropriate age. District-specific population age-distribution and birth rate data came from the 2000 Population and Household Census district-specific data, adjusted to 2010 values by a 1.45% annual population growth rate [7]. The Thai National Health Statistic Office (NHSO) provided monthly patient arrival data to adjust our projections. All arrival rates were sub-divided into target age groups (newborns, 0–1 year, 1–2 years, 2–3 years, 3–4 years, and pregnant women) by the proportion of the total population represented by each sub-group from the 2000 census and the number of visits each age group needs to make for their respective vaccination schedule. Newborns presented only to hospitals (i.e., district level locations). Based on a previous study in Thailand, 90% of children sought vaccination in public clinics (versus private locations) [8]. The amount of diluent that is stored in the refrigerator the day prior to administration is dependent on the rate of patient arrival.

### Vaccine Wastage

 The models account for three kinds of vaccine loss throughout the vaccine supply chain: shipping, inventory, and open vial loss. Shipping and inventory loss results from breakage or temperature exposure. Open vial loss accounts for the incomplete consumption of full vials (e.g., only 3 doses used from a 5-dose vial) as described by previous studies and the WHO's Multi- Dose Vial Policy [2], [9]. Closed vial wastage rates for each vaccine are input into the model and account for breakage during transport and storage. It is difficult to estimate closed vial wastage (i.e., wastage of vaccine before a vial is opened) in shipping and storage because these rates are seldom reported separately from open vial waste and may sometimes stem from vaccine mismanagement [10]. Communications with vaccine logistics experts suggested 1% inventory and shipping loss rates. We then additionally explored 2% and 3% loss rates in sensitivity analyses. Additionally, vaccine temperature profiles were input into HERMES to track exposure to adverse temperatures. When a vaccine is stored outside its preferred temperature range (e.g., when there is not enough space in a cold storage device), it is stored at room temperature, and expires at a faster rate.

### Vaccine Characteristics

The supply chain in Trang Province contains seven current vaccines whose total packaged volume per fully immunized child (FIC) is 149.7 cm^3^. Vaccine volumes include volumes of the vaccine, the vaccine vials and the packaging material, which vary by presentation and in some cases can be rather bulky [11]. Table 1 lists characteristics of current EPI vaccines as well as non-EPI vaccines included in our introduction scenarios.

**Table 1:** Thailand NIP Vaccine Characteristics.

Vaccine	Status in National Immunization Program (NIP)*	Doses per vial	Packaged volume per dose (cm3)	Administration Schedule as per WHO immunization profile	Temperature Profile (preferred storage medium)	Sources
Bacille Calmette-Guerin (BCG)	NIP vaccine	10	1.2	Birth	12 months at 2–8°C	[34], [35], [36]
Hepatitis B (HepB)	NIP vaccine	2	13.0	Birth	36 months at 2–8°C	[34], [35], [36]
Diphtheria-tetanus-pertussis (DTP)	NIP vaccine	10	3.0	1.5–2 years and 4–5 years	18 months at 2–8°C	[34], [35], [36]
Diphtheria-tetanus-pertussis-hepatitis B (DTP-HepB)	NIP vaccine	10	3.0	2,4 and 6 months	36 months at 2–8°C	[34], [35], [36]
Oral polio vaccine (OPV)	NIP vaccine	20	1.0	2,4,6,18 months and 4–5 years	12 months at 0 to −15°C, 1 month at 2–8°C	[34], [35], [36]
Measles (M)	NIP vaccine	10	3.5	9 months	24 months at 2–8°C	[34], [35], [36]
Japanese encephalitis (JE)	NIP vaccine	2	12.6	1.5–2 years (twice), and 2.5–3 years	24 months at 2–8°C	[34], [35], [36]
Seven-valent pneumococcal conjugate vaccine (PCV-7)	Non-NIP vaccine for experimental introduction	1	55.9	2,4,6 and 15 months	24 months at 2–8°C	[35], [36], [37]
Rotavirus (RV)	Non-NIP vaccine for experimental introduction	1	17.1	2 and 6 months	24 months at 2–8°C	[35], [36], [38]
Rotavirus (RV)	Non-NIP vaccine for experimental introduction	1	45.9	2,4 and 6 months	24 months at 2–8°C	[35], [36], [38]
Rotavirus (RV)	Non-NIP vaccine for experimental introduction	1	79.8	2,4 and 6 months	24 months at 2–8°C	[35], [36], [38]
Rotavirus (RV)	Non-NIP vaccine for experimental introduction	1	156.0	2 and 6 months	24 months at 2–8°C	[35], [36], [38]
Rotavirus (RV)	Non-NIP vaccine for experimental introduction	1	259.8	2 and 6 months	24 months at 2–8°C	[35], [36], [38]

*School aged children and pregnant women were not considered in our analysis.

### Sensitivity analyses

Table 2 lists model inputs used in our simulations. Sensitivity analyses systematically explored the effects of ranging the following parameter values: random inventory loss rate, i.e., percentage of vials damaged during storage (range: 1–3%), shipping loss rate, i.e., percentage of vials damaged during shipping (range: 1–3%), refrigerator capacity utilization, i.e., the percentage of refrigerator space that can actually be used to store vaccines (range: 50–100%), and shipping frequencies (twice per month, once per month and once every two months). In addition, we varied the population demand between a static (i.e., number of patients in a month is fixed based on projected population estimates and does not fluctuate from month to month) versus stochastic monthly distribution [i.e., number of vaccine recipients in a given month draws from a Poisson distribution with a mean of (λ)], and the birth rate in the target population to be vaccinated (range: 95–105% of projected 2010 vaccine recipients).

**Table 2:** Model Parameters and Ranges for Sensitivity Analyses.

Variable	Baseline value	Range or Alternative
***Demand***		
Annual population growth rate (%)	1.45	None
Number of newborns per year	10,910	10,365–11,456
0–1 year olds per year (excluding newborns)	26,778	25,439–28,117
2–3 year olds per year	26,778	25,439–28,118
3–4 year olds per year	26,778	25,439–28,119
Population distribution across months	Stochastic	Fixed
***Supply Chain Network and Policies***		
Closed vial wastage per storage period (%)	2%	1–3%
Closed vial wastage per shipment (%)	2%	1–3%
Requisition, procurement and delivery schedules (interval between events in number of days)	30	15–60
***Cold Chain Equipment***		
Cold truck capacity (liters)	6,480	None
Cold box capacity (liters)	19	None
Vaccine carrier (liters)	5	None
Regional level cold storage capacity (liters in refrigerator)	29,461	17,330–34,660
Provincial level cold storage capacity (liters in refrigerator)	560	330–659
District health office cold storage capacity (liters in refrigerator)	638	375–750
District municipal health center cold storage capacity (liters in refrigerator)	145	86–171
District hospital cold storage capacity (liters in refrigerator)	364	214–428
Sub-district cold storage capacity (liters in refrigerator)	119	70–140
Regional level cold storage capacity (liters in freezer)	231	136–272
Provincial level cold storage capacity (liters in freezer)	349	205–410
District health office cold storage capacity (liters in freezer)	11	7–13
District municipal health center cold storage capacity (liters in freezer)	65	39–77
District hospital cold storage capacity (liters in freezer)	167	99–197
Sub-district cold storage capacity (liters in freezer)	21	13–25

### Supply Chain Performance Measures

The overall objective of the EBM is to maximize the mean vaccine supply ratio (i.e., a measure of vaccine availability for patient arriving to get immunized) across all immunization locations over the course of a year. The supply ratio is a measure of the number of arriving patients who receive vaccination because there is enough vaccine in stock and is computed in the EBM as follows:
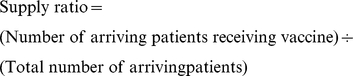
The constraints are the storage and transport capacities of each location and the shipping frequencies as outlined above. The EBM focused on maximizing supply ratio without considering costs.

The main purpose of the EBM was to compare and cross-validate HERMES. Although the EBM may be used as an optimization model, the goal of this study was to simulate new vaccine introduction while accounting for the stochastic nature of many of the parameters. Therefore, all of the results reported from here on come from experiments performed with HERMES. We also validated our HERMES model by comparing simulation output with observations from the field in Thailand (e.g., prior to new vaccine introduction, the same locations that demonstrated capacity constraints in HERMES experienced capacity constraints in real-life).

## Results

### Overall Impact

Introducing the new vaccines could clog up the refrigerator space at the provincial and district levels and the transport cold space at all levels thereby preventing other vaccines from reaching the clinics and being available to patients who arrive to be immunized. The new vaccine introduction decreases the supply ratio (i.e., the percentage of people arriving at a clinic to be immunized who subsequently have vaccines available to them). Only the smallest vaccine (the 17.1 cm^3^/dose RV) would have no appreciable effect on vaccine availability at clinics. All other RV and PCV-7 vaccines are too large for the current supply chain to handle without modifications such as increasing storage or transport capacity or shipping frequency.

Our findings were robust to varying shipping and inventory loss rates (range: 1–3%), storage capacity utilization (85% and 100%), and target population size (range: 95–105%). Switching from a stochastic to a fixed monthly population distribution also did not statistically significantly alter vaccine supply ratios across vaccine introduction scenarios (i.e., their 95% confidence intervals overlapped). However, reducing storage capacity utilization to 50%, and to reducing vaccine shipping frequency to every 30 days and every 60 days did have significant effects on the supply ratios. Therefore, from here on we report only results from scenarios that employ 1% random inventory and shipping loss with a monthly shipping policy and utilizing stochastic demand.

### Impact on Storage Facilities

**Figure 2:**
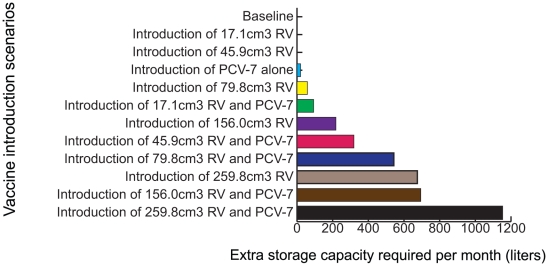
Extra Storage Capacity Required at the Provincial Store Following Each Vaccine Introduction Scenario (Results from HERMES).

**Figure 3:**
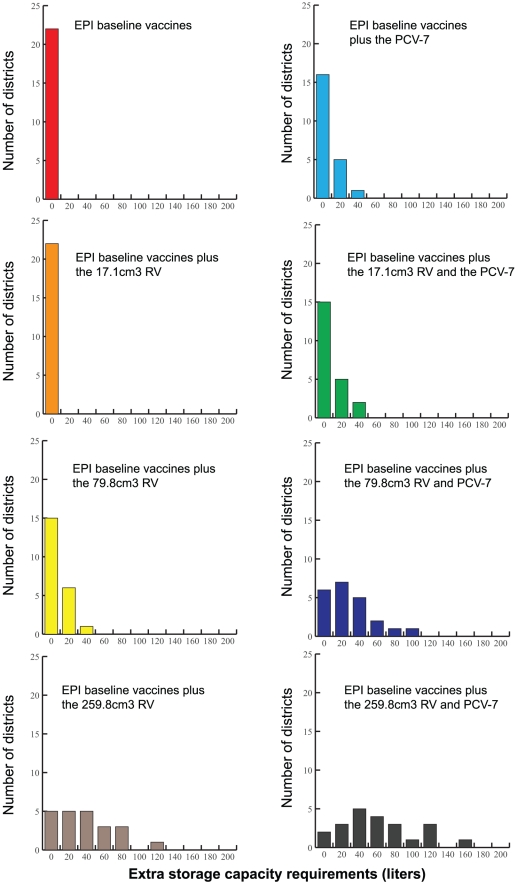
Extra Storage Capacity Required at the District Level Following Various Vaccine Introduction Scenarios (Results from HERMES).

Introducing the new vaccines exceeds available refrigerator space for the largest vaccine sizes, exceeds available refrigerator space for all but the smallest two presentations alone (i.e., 17.1 and 45.9 cm^3^/dose) at the provincial level, and all but the smallest presentation at the district level. All other presentations could overwhelm some district level locations' storage capacities. Figure 2 shows the additional cold storage capacity needed at the provincial level and Figure 3 shows the additional cold storage capacity required at the district level following various RV and PCV-7 introduction scenarios. The need for more storage is greater when shipping frequency decreases, which could happen with the break down or decommissioning of vehicles, loss of personnel, or political, weather, or financial problems. For example, dropping the shipping frequency to bi-monthly (once every two months) would require an increase in storage capacity for all but the two smallest vaccines at the provincial level and all presentations at the district level.

### Impact on Transport Capacity

**Figure 4:**
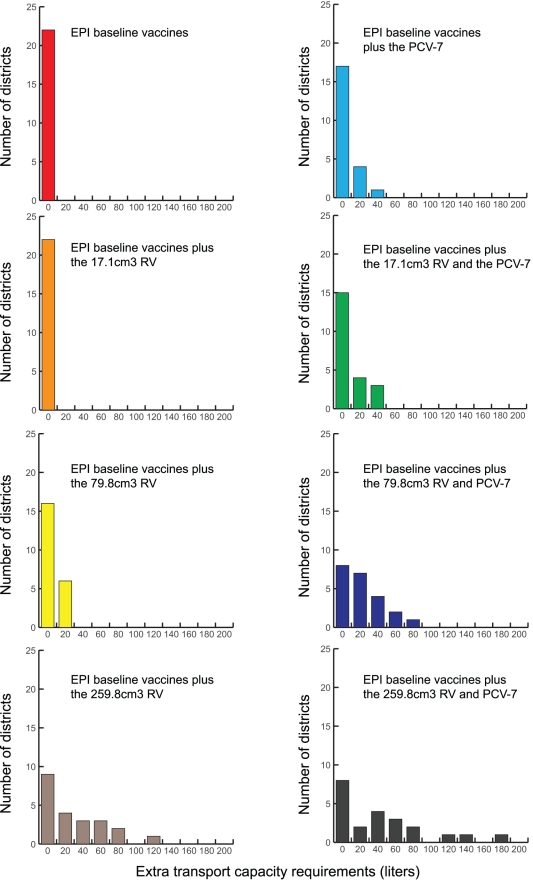
Extra Transport Capacity Required at the District Level Following Various Vaccine Introduction Scenarios (Results from HERMES).

**Figure 5:**
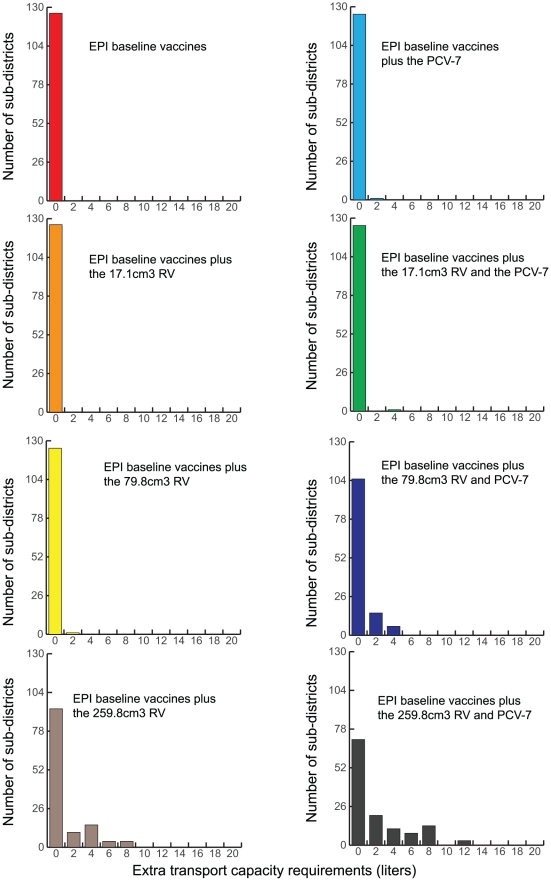
Extra Transport Capacity Required at the Sub-district Level Following Various Vaccine Introduction Scenarios (Results from HERMES).

Transport cold capacity is a limitation as well. Current transport cold capacity can only fully handle the two smaller (17.1 cm^3^/dose or the 45.9 cm^3^/dose) RV presentations. No additional transport capacity is required when delivering vaccines from the regional to the provincial level; however, as figures 4 and 5 show, increases in capacity are needed from the district and sub-district levels. Decreasing shipping frequency exacerbates this need.

### Impact on Vaccine Administration

**Figure 6:**
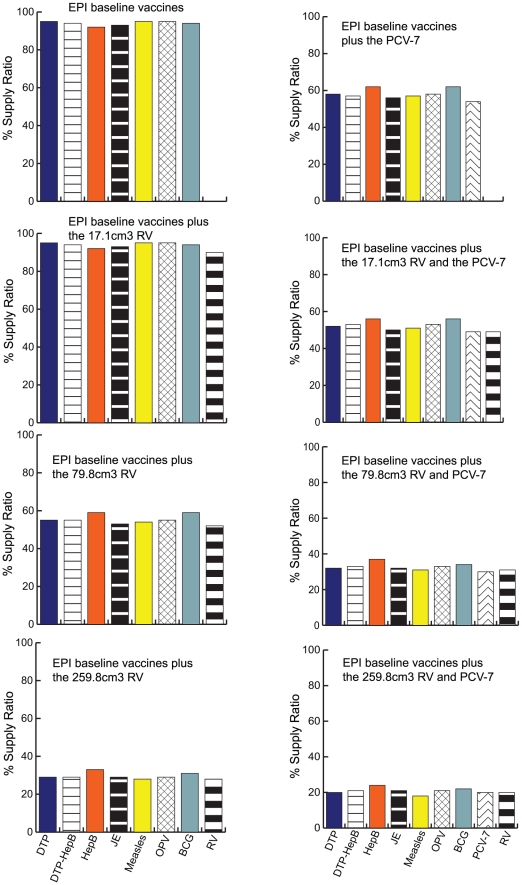
Frequency Histogram of Vaccine Supply Ratio for District and Sub-district Locations (Results from HERMES).

The storage and transport bottlenecks inhibit the flow of all EPI vaccines to many of the health clinics, thereby decreasing vaccine availability for patients. Currently, the supply ratio for sub-districts is 100%, i.e., everyone arriving at a clinic can get immunized. Figure 6 shows how the supply ratios drop as the size of the new vaccines increase. The impact on health clinics is widely variable. Some sub-districts are able to maintain close to 100% supply ratios. Others experience 10 to 15% drops, while others suffer even more dramatic declines (down to 18% for the largest RV plus PCV presentations).

Again these declines would be even more dramatic if the current shipping frequency could not be maintained. Decreasing the shipping frequencies to bi-monthly would result in supply ratios ranging from close to 100% for smaller presentations, down to an average of 5% for the largest vaccine size combination.

Vaccine availability across the year following vaccine introduction varied slightly showing natural stochasticity, but because we evaluated routine immunization with RV and PCV rather than campaign or roll-out strategies for immunization, the variations in vaccine availability from one month to the next were not dramatic.

The variability of supply ratios and additional storage and transport capacities that exists between districts and between sub-districts is largely accounted for by the differences in population demand at each site and at a given immunization session.

## Discussion

Our results highlight the importance of evaluating the complex system-wide effects of introducing a new vaccine into a vaccine supply chain. Vaccine distribution is a complex process and any change in the vaccines that flow through the supply chain may have untoward ramifications. Supply chains may not be able to accommodate the introduction of new vaccines and in turn delay the delivery of new and current vaccines to arriving patients. When developing a vaccine, vaccine scientists and manufacturers should account for the impact of a vaccine's presentation size. Moreover, prior to a new vaccine introduction, public health decision makers may want to review a country's supply chain structure and operations and evaluate possible adaptations; otherwise, complications can be substantial.

Results from our model can be utilized in several ways. It can help determine whether a supply chain is able to handle one or multiple vaccine introductions. It can also help guide vaccine presentation selection when different presentations are available. Even when only one presentation is available, our model can help identify where decision makers need to expand capacity (e.g., procure more trucks or refrigerators).

The use of computational models can be helpful in planning new vaccine introduction and identify potential shortcomings well in advance. Decision makers can make use of models to understand complex repercussions that may not be outwardly obvious. The use of computational models, while limited in public health [12], [13], [14], have been used widely in many other industries (e.g., manufacturing and distribution [15], transport [16], aerospace [17], and military and defense [18]) as logistics planning tools. Recent public health research using computational models have helped evaluate various infection control measures [19], [20], [21], [22], [23], [24], [25], [26], [27], [28]. The Department of Health and Human Services developed large scale computational models in the response to the 2009 H1N1 pandemic [19], [20], [22], [29], [30]. There are some examples in the literature of computational models of perishable or temperature sensitive products such as food processing and distribution [31]. Developing and implementing computational models could save much time, expenses, and effort in vaccine supply chain management, help inform decisions about vaccine introductions, save costs and ensure adequate vaccination of children. These advancements could lead to significant public health benefits.

### Limitations

By definition, models are simplifications of real life and cannot account for every possible factor, relationship, or outcome [32], [33]. Substantial data collection from a wide variety of different sources was necessary in the construction of our model. Thailand is currently in a transition to a new Vendor Managed Inventory system. Some data used to construct the model comes from the previous system. For example, the newer supply chain distributes vaccines from the distributor to the hospitals directly which did not occur in this model. Other data such as the availability and size of transport vehicles may not be as reliable, since equipment inventories are subject to change. Furthermore, random events such as power outages were not captured for in our model. Our experiments assumed that new vaccine introduction would occur immediately and not gradually over a period of time. Rather than visit every single location in the supply chain, our team sampled sets of locations and extrapolated measurements to comparable locations. We assumed that 60% of individuals seek immunization at district locations and 40% at sub-district locations. Our model assumed that shipments would occur as scheduled. Finally, vaccination demand at each district and sub-district was estimated from census data and may vary in real life. Nevertheless, performing sensitivity analyses indicated our results to be fairly robust to changing parameter values. Actually, several of the limitations (e.g., random disruptions to the supply chain) would likely further reduce vaccine availability after RV or PCV-7 introduction.

### Conclusion

Introducing the currently available formulations of PCV-7 and/or RV vaccines to the routine EPI vaccine supply chain in Thailand could substantially inhibit the delivery of all current EPI vaccines. Therefore, considerable planning and modifications may be necessary prior to the introduction of new vaccines. This study emphasizes the importance of considering the effects on the entire vaccine supply chain when introducing a new vaccine and how computational models can assist public health officials, manufacturers, and other key decision makers plan for new vaccine introduction.
